# The Poultry-Associated Microbiome: Network Analysis and Farm-to-Fork Characterizations

**DOI:** 10.1371/journal.pone.0057190

**Published:** 2013-02-27

**Authors:** Brian B. Oakley, Cesar A. Morales, J. Line, Mark E. Berrang, Richard J. Meinersmann, Glenn E. Tillman, Mark G. Wise, Gregory R. Siragusa, Kelli L. Hiett, Bruce S. Seal

**Affiliations:** 1 Poultry Microbiological Safety, USDA Agricultural Research Service, Richard B. Russell Agricultural Research Center, Athens, Georgia, United States of America; 2 Bacterial Epidemiology and Antibiotic Resistance Research Unit, USDA Agricultural Research Service, Richard B. Russell Agricultural Research Center, Athens, Georgia, United States of America; Missouri University of Science and Technology, United States of America

## Abstract

Microbial communities associated with agricultural animals are important for animal health, food safety, and public health. Here we combine high-throughput sequencing (HTS), quantitative-PCR assays, and network analysis to profile the poultry-associated microbiome and important pathogens at various stages of commercial poultry production from the farm to the consumer. Analysis of longitudinal data following two flocks from the farm through processing showed a core microbiome containing multiple sequence types most closely related to genera known to be pathogenic for animals and/or humans, including *Campylobacter, Clostridium*, and *Shigella*. After the final stage of commercial poultry processing, taxonomic richness was ca. 2–4 times lower than the richness of fecal samples from the same flocks and *Campylobacter* abundance was significantly reduced. Interestingly, however, carcasses sampled at 48 hr after processing harboured the greatest proportion of unique taxa (those not encountered in other samples), significantly more than expected by chance. Among these were anaerobes such as *Prevotella*, *Veillonella*, *Leptrotrichia*, and multiple *Campylobacter* sequence types. Retail products were dominated by *Pseudomonas*, but also contained 27 other genera, most of which were potentially metabolically active and encountered in on-farm samples. Network analysis was focused on the foodborne pathogen *Campylobacter* and revealed a majority of sequence types with no significant interactions with other taxa, perhaps explaining the limited efficacy of previous attempts at competitive exclusion of *Campylobacter*. These data represent the first use of HTS to characterize the poultry microbiome across a series of farm-to-fork samples and demonstrate the utility of HTS in monitoring the food supply chain and identifying sources of potential zoonoses and interactions among taxa in complex communities.

## Introduction

As the source of a majority of emerging infectious diseases, animal-associated microbiomes represent a nexus of food safety, animal health, and public health [1], [2], [3]. The poultry microbiome is of particular interest as a major source of foodborne infections both worldwide and in the U.S., where foodborne pathogens are estimated to have accounted for 47 million episodes of illness at an economic cost of $77 billion in 2011 [4], [5]. In the course of commercial poultry production, chickens typically progress in a single age cohort from a microbiologically depauperate gastrointestinal (GI) tract in newly-hatched chicks to market age adults at 6–8 weeks harbouring up to 10^11^ bacterial cells g^-1^ of intestinal contents [6], representing hundreds [7], [8] to thousands [9] of distinct taxa. The poultry GI microbiome differs ecologically from mammals in that colonization occurs primarily from the surrounding environment and individuals of the same age reared in close proximity rather than through direct contact with adults [10], [11], [12], [13], [14], [15]. Colonization of poultry by microbes from environmental sources associated with commercial poultry production may have important biosecurity and management implications if human pathogens are transferred from environmental reservoirs through the poultry supply chain to consumers. This potential is recognized in the recently implemented Food Safety Modernization Act which emphasizes prevention of foodborne illness via monitoring of the entire food supply chain [16], and thus serves as a compelling mandate for a microbial census along what has been termed the ‘farm-to-fork’ continuum.

Detection and enumeration of poultry-associated bacteria at various stages of commercial poultry production have been the subject of several decades of research, generally focused on known pathogens such as *Campylobacter*. For example, transmission of *Campylobacter* between sequential flocks has been demonstrated [17], [18], , and specific *Campylobacter* genotypes present on farms have been linked to human illness [21], [22]. Although such research has answered important questions, pathogen surveys have typically relied either on cultivation of a specific organism of interest or molecular assays designed from strains already in cultivation - effectively limiting such surveys to the small proportion of microbial diversity recoverable by cultivation [23]. High-throughput sequencing (HTS) offers a solution to this previously intractable problem by rapidly taking a census of a microbial community independently of the ability to culture resident organisms. Although the exact magnitude of taxonomic richness that is actually measured by current sequencing technologies is still a matter of some debate [24], [25], [26], the fact that only a small proportion (<1%) of microbial taxa are cultivable as first noted by Staley and Konopka [27] remains true today. For human pathogens, fewer than half of clinically important pathogens are thought to have been positively identified by cultivation [2], [28], and unknown agents are estimated to cause four times as many episodes of foodborne illness as 31 major known pathogens [29]. HTS is an important new tool to identify emerging pathogens and explore previously uncharacterized microbial habitats.

As sequencing throughput has continued to increase and costs decrease in recent years, an opportunity has been created to greatly expand on previous characterizations of the poultry gut microbiome [30], [31], [32], compare communities, and identify specific taxa throughout the farm-to-fork continuum of poultry rearing and consumption. HTS-based characterizations of microbial communities along the poultry supply chain will help to provide a baseline census, document the dynamics of known pathogens, identify emerging pathogens, and help target specific interventions to improve animal and human health. HTS data sets are also highly amenable to network analysis which can offer novel insights into community composition, taxonomic interactions, and ecological niche associations in complex microbial communities [33], [34], [35].

In the current study, we combined 454 and Illumina sequencing of 16S rRNA gene amplicons with quantitative-PCR focused on putative pathogenic organisms and virulence genes to compare bacterial community structure and abundance of specific pathogens from poultry fecal samples, litter samples, carcass rinses, and carcass weeps, including the metabolically active bacterial community from fresh poultry products purchased at retail. For a subset of the samples, two flocks were followed longitudinally from rearing through processing. Our main objectives were to answer the following questions: 1) Which taxa are shared between bacterial communities in the litter of typical commercial poultry houses and the birds themselves? 2) How similar are these communities to those associated with chicken carcasses following commercial processing? 3) How does the carcass-associated community change during shelf-storage conditions? 4) what bacterial taxa are associated with poultry products as typically sold to the consumer?, and 5) what taxa, if any, are significantly associated with the foodborne pathogen *Campylobacter*?

## Materials and Methods

### Sample Collection

#### Longitudinal samples

Initial collections of litter and fecal samples were made in October 2010 and February 2011 from two commercial broiler chicken production houses in the southeastern U.S., each containing a single flock of ca. 16,000 birds (Ross x Hubbard) six weeks of age. The flocks were selected to represent typical industry practices which include the administration of sub-therapeutic amounts of antibiotic growth promoters (AGPs). Litter (pine shavings) and fresh fecal droppings were collected to be representative of the entire flock by collecting from 25 locations within a semi-regular grid covering each poultry house. Samples were pooled into five sets of five, diluted 1∶3 (w:v) with 1× PBS, homogenized for 1 min, and 400 µl of the filtrate from sterile blending bags with full-width membrane filters (Model R36840-56, Cole Parmer, Vernon Hills, IL) used for DNA extractions as described below. Litter was collected as dry samples with ambient moisture content and wet samples saturated with water from directly underneath drip lines. Samples were stored at 4°C and transported to the laboratory and processed within 6 hours of sampling. DNA extractions were performed on each of the five sub-samples according to the protocol for pathogen detection from human stool with the QIAamp DNA Stool Kit (Qiagen, Valencia, CA, USA). For each sample type (fecal, wet litter, dry litter), the five sub-samples were normalized and pooled after DNA quantification to form a single sample for each poultry house.

Two weeks after initial sample collection, at ca. 8 weeks of age, these same flocks were commercially processed per typical industry practices including the immersion of carcasses in chlorinated water as the final step in processing. Following processing, 25 carcasses were collected from the chlorinated chill tank, aseptically bagged, transported to the lab, and shaken in an automated shaker [36] for 2 min after addition of 100 mL of sdH_2_O. The resulting fluid (hereafter referred to as ‘carcass rinse’) was collected aseptically. After remaining in the same bags for 48 hr at 4°C, an additional 50–75 mL (hereafter ‘carcass weep’) was collected from each carcass. For both carcass rinse and weep samples, samples from all 25 birds were pooled in sets of five, concentrated to ca. 2 mL by centrifugation and DNA extractions performed as above with the QIAamp DNA Stool Kit (Qiagen, Valencia, CA, USA) using 200 µL of the cell suspension. For each sample type (carcass rinse, carcass weep), the five sub-samples were normalized and pooled as above to form a single sample.

#### Retail samples

To characterize the microbial community with potential metabolic activity (defined by the presence of intact rRNA molecules) in poultry products as typically purchased by consumers, poultry packages were purchased at retail in April 2012 and total nucleic acids extracted using the Griffiths method [37]. Aqueous exudate was collected directly from six packages of uncooked chicken parts (drumsticks, thighs and breasts); each package included multiple parts from chicken flocks processed in different slaughter plants on different dates from three different producers. From each package, 3 mL of exudate (hereafter ‘retail weep’) was collected, filtered through a coarse membrane filter as above, and centrifuged at 10,000 g for 10 min. Nucleic acid extractions were re-suspended in 35 µL of DEPC-treated TE, and incubated with DNase (Promega M6101) at 37°C for 30 min followed by a 10 min denaturation at 65°C per manufacturer’s recommendations. Subsequently, 2.75 µL of this reaction was incubated with 5 pmol of the primer 530R (5′- CCGCNGCNGCTGGCAC - 3′) at 70°C for 5 min and reverse-transcribed by incubation with random hexamers and reverse transcriptase (Invitrogen Superscript II) at 42°C for 50 min per manufacturer’s recommendations. PCR was performed with the primers 104F (5′-CCGCNGCNGCTGGCAC-3′) and 530R targeting the V1–V3 region of the 16S rRNA gene as previously described [8]; PCR products representing retail weep fluid from six packages were pooled for sequencing. For all amplifications of cDNA, corresponding negative results were confirmed for DNase treated samples without reverse transcription.

#### Samples for network analysis

Samples collected for the longitudinal study described in detail above were combined with samples previously collected by our research group to build an adequate data set for network analysis. The previously collected samples were comprised of cecal contents (pooled from five individuals each) from 1, 2, and 3 week-old birds as described [38]; cecal samples from 3 week-old birds [39]; and carcass rinses collected during commercial processing as previously described [40]. Each sample was screened to ensure it was *Campylobacter* positive.

No specific permits were required for the described studies. Verbal permission to collect samples for this study was obtained from the commercial owners of private property in Georgia. Sample collection did not involve endangered or protected species. Additional details for all samples are provided in Table S1.

### Sequencing and Data Analysis

PCR and 454 pyrosequencing were performed by Research and Testing Laboratory (Lubbock, TX) and USDA Agricultural Research Service (Athens, GA) using the same primers as above and tagged amplicon methods as previously described [8], [41]. Sequences were processed in mothur [42] using quality files with a 50 bp moving window at an average quality score cutoff of 35 and a maximum number of homopolymers below eight. Further processing per recent recommendations [24], [25], [43] and standard protocols (http://www.mothur.org/wiki/Schloss_SOP) was completed using Perl and Bioperl scripts to trim pyrosequencing tag sequences, screen for presence of the forward PCR primer sequence, and remove sequences with any ambiguous base calls. Based on expected amplicon sizes and frequency distributions of sequence lengths in v104 of the Silva reference database [44], sequences were further limited to a range of 325–425 bp. Sequences which passed these screens were then aligned to the Silva reference dataset using PyNAST [45] implemented in QIIME [46] and putative chimeric sequences were identified with ChimeraSlayer in mothur [42].

Illumina HiSeq2000 sequencing was performed using two flow cell lanes to generate paired-end reads from PCR products of the 16S rRNA V3 region obtained with the primers 341F (5′- CCTACGGGNGGCWGCAG-3′) and 519R (5′- ATTACCGCGGCTGCTGG-3′). from the 14 cecal and carcass rinse samples collected previously [38], [39],[40] as detailed above and in Table S1. Paired-end reads were merged with flash [47] and processed with the fastx toolkit [48] in a command line implementation with default settings. After random sub-sampling of 20,000 sequences per sample, the merged and quality-trimmed reads were trimmed to between 160 and 190 bp in length. The effects of the shorter Illumina reads on taxonomic classification and clustering was determined by trimming 454 reads to the same region (160–190 bp upstream of the 341F primer) and comparing to untrimmed reads of the same set of sequences. The misclassification rate determined by this approach was consistent with previous observations [49], and well within the acceptable range for the analysis presented here, as no *Campylobacter* sequences were misclassified. Rarefaction curves for all samples at each of three similarity cutoffs are shown in Figure S1.

Taxonomic classifications were based on the EMBL taxonomy from the Silva project (v104) curated seed database [44] using usearch [50] with the global alignment option. To assess phylotype richness and diversity independent of taxonomic classifications, sequences which passed all the screens described above were grouped into similarity clusters (operational taxonomic units; OTUs), using similarity cutoffs of 90%, 95%, and 97%. As a methodological comparison, both CD-HIT [51] and usearch [50] were used, both run with default parameters. Using control data sets derived from pyrosequencing of single colonies, we have previously shown CD-HIT to be a much more conservative clustering algorithm than approaches based on distance-matrices derived from multiple sequence alignments [41], which are known to falsely inflate richness and diversity estimates [25]. The output from CD-HIT and usearch provided the inputs for a data analysis pipeline we constructed to parse the clustering results, provide input for mothur [42], and produce graphical and statistical summaries of the data for the desired sampling units using R [52]. More information and open-source code can be found at http://go.warwick.ac.uk/thermophyl/pipeline. Sequence data have been deposited in GenBank with BioSample accession numbers: SAMN01853131–SAMN01853156 and MG-RAST as ID 4511219.3–4511244.3.

Network analysis was conducted as previously described [34] using normalized OTU tables at various levels of clustering and removing OTUs or taxa represented by fewer than five sequences or <0.5% total relative abundance across all samples. Spearman correlation coefficients of 0.7 and p-values of 0.001 were required to establish valid co-occurrence among OTUs. Network analysis was performed in R with the igraph package and visualized with the program Gephi.

### Quantitative PCR

Quantitative PCR for 16S rRNA genes was performed with SYBR green chemistry (ABI, Foster City, CA, USA) using primer sets previously validated as specific for Clostridial Group I [53] and the genus *Campylobacter* [7] with thermal cycling protocols as previously described [53]. PCR products were obtained from *Clostridium perfringens* ATCC 13124 and *Campylobacter jejuni* NCTC 11168 and purified with Qiaquick spin columns (Qiagen, Valencia, CA, USA). The purified products were subsequently cloned into vector pCR4-TOPO using the TOPO TA Cloning Kit (Invitrogen, Carlsbad, CA, USA) following manufacturer’s recommendations and used as standards for absolute quantification for each respective assay.

To target *C. jejuni* specifically, a new probe and primer set targeting the beta-subunit of the cytolethal distending toxin gene (*cdt*B) was designed with the program ThermoPhyl [54] using publicly available sequences retrieved with BioPerl from Genbank, and assay sensitivity and specificity were confirmed with ARB [55]. ABI TaqMan chemistry was used with standards as above and universal cycling conditions per manufacturer’s recommendations. For all reactions, R^2^ values of standard curves were at least 0.98 and reaction efficiency was between 90–110%.

## Results and Discussion

### Longitudinal Farm-to-fork Data Set

#### Comparisons of core microbiome

**Figure 1:**
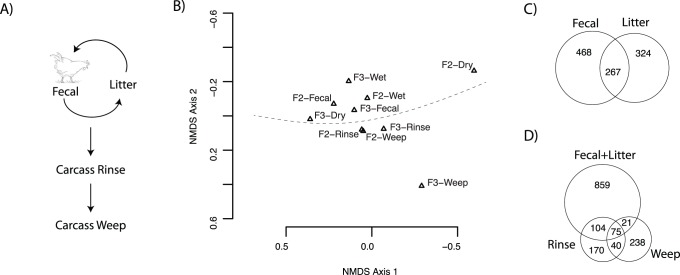
Overview of sample types collected and similarity of samples across the farm-to-fork continuum. A) Sample types collected with a simplified model of microbial community interactions. B) Non-metric dimensional scaling representation of overall community similarity of sample types based on 3% OTU abundance. Samples represent two farms (F2, F3) with litter samples split into dry and wet litter. C) Number of shared and unique OTUs between the fecal and litter microbiomes at a 3% cutoff. D) Shared and unique 3% OTUs among fecal and litter samples, carcass rinse, and carcass weep samples. For both (C) and (D), Venn diagrams are scaled according to OTU membership with singleton and doubleton OTUs excluded. Line drawing is from http://www.allaboutdrawings.com/bird-drawings.html.

To address the potential for microbes from the poultry-rearing environment to be transferred through the poultry supply chain to consumers, we followed two flocks of ca. 16,000 birds each from the farm through poultry processing, collecting samples from the chickens and their immediate environments. These samples included fecal, litter, and carcass rinse and weep samples as detailed in the methods section and Table S1. As a simplified conceptual model of community interactions, we hypothesized that a large proportion of taxa would be shared between the litter and the fecal samples, and additionally, that the majority of sequences recovered from carcass rinses and subsequent weeps should represent a subset of those present in the litter and fecal samples (Figure 1A). These hypotheses were based on the following observations (reviewed in [12], [14], [56]: 1) newly-hatched chicks have a naïve intestinal community, 2) chickens are hunt-and-peck feeders, frequently contacting their litter, 3) chickens are coprophagous, 4) litter from previous flocks with a well-developed microbial community is commonly re-used, and 5) poultry houses are confined environments in which birds are exclusively reared after ca. 2 d of age until harvest 6–8 weeks later. Additionally, our fecal samples were collected as fresh droppings - because they did have some contact with the litter, overlap between the fecal and litter communities should be expected. Based on all of these factors, we expected to observe well-mixed microbial communities with many taxa shared across sample types.

Contrary to these expectations, neither hypothesis above was well supported. UniFrac analysis rejected a null hypothesis of no phylogenetic clustering by sample type (p<0.001), and fecal and litter samples generally clustered with each other as distinct from the rinse and weep samples in ordinations representing overall community similarity (Figure 1B). Moreover, each of the fecal, litter, carcass rinse, and carcass weep samples contained more unique OTUs than OTUs shared with any other sample type, even when uncommon sequence types were excluded from consideration (Figure 1C, 1D). Poultry-associated microbial communities along the farm-to-fork continuum clearly have major differences in community structure. We expected to find differences in relative abundance of various taxa among the sample types, but the extreme differences observed for taxon presence/absence were surprising. Ecological mechanisms such as dispersal limitation, differences in colonization ability, habitat filtering, niche exclusion, or competition may contribute in varying degrees to the observed differences in community structure and some (such as niche exclusion) may offer potential to suppress specific human pathogens at various stages of the poultry production process.

**Figure 2:**
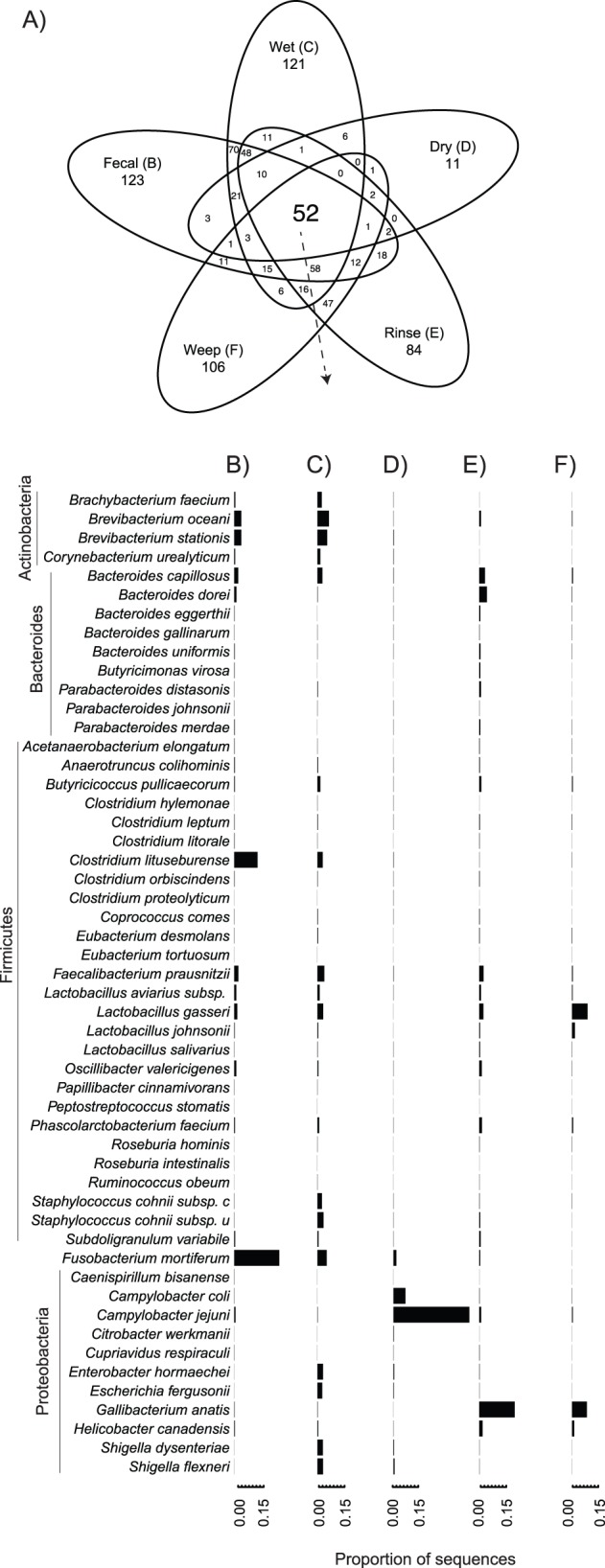
Core microbiome as classified to the species level with usearch against the Silva reference database as described in the methods. A) Number of shared and unique taxa for each of the five sample types shown. The relative abundance of the 52 taxa belonging to a core microbiome common to all samples is shown for B) Feces, C) Wet litter, D) Dry litter, E) Carcass Rinses, and F) Carcass Weeps.

To determine in more detail the nature of the shared (core) versus unique (satellite) bacterial community by sample type, we next performed a taxonomic classification of sequences against a reference database using both blastn and usearch as described in the methods. The two methods gave very similar results with only minor discrepancies (data not shown). We concluded that either method is appropriate although usearch has a significant advantage of speed (in our tests, nearly 300× faster than blastn run locally). At a species level, a core set of 52 taxa were common to all sample types (Figure 2A). Within this core group, the most abundant sequence types in the fecal sample were most closely related to the anaerobic gram-negative *Fusobacterium*, gram-positive Actinomycete genera such as *Brevibacterium*, *Bacteroides*, and the Firmicute genera *Clostridium*, *Faecalibacterium*, and *Lactobacillus* (Figure 2B), consistent with previous observations [9], [31], [57], [58], [59]. For the wet litter samples, the relative abundance of these taxa was fairly similar to the fecal samples, with the exception of higher proportions of sequences most closely related to *Staphylococcus* and sequences most closely related to *Shigella* (Figure 2C). *Shigella* has not generally been considered to be associated with poultry [60], but our results are consistent with other observations [32], [61] and may expand the list of potential poultry-associated pathogens.

In contrast to the general similarity of the fecal and wet litter samples, one of the dry litter samples was unusual in its domination by sequences most closely-related to *C. jejuni* (Figure 2D). *Campylobacter* has been shown to quickly lose viability when inoculated onto fresh, dry litter [62], and although DNA-based PCR can amplify 16S rRNA genes from dead cells, it is difficult to argue against *C. jejuni* as abundant in the dry litter either at the time of sampling or in the relatively recent past given the predominance of these sequences in this sample type (Figure 2D). Core taxa detected in the carcass rinse and weep samples were generally similar in their relative abundance (Figure 2E, 2F). These two sample types were distinguished from the fecal and litter samples by several relatively abundant taxa, notably *Gallibacterium anatis*, strains of which have been shown to cause several diseases in poultry including peritonitis, salpingitis, and septicemia [63].

#### Comparisons of satellite microbiome

**Figure 3:**
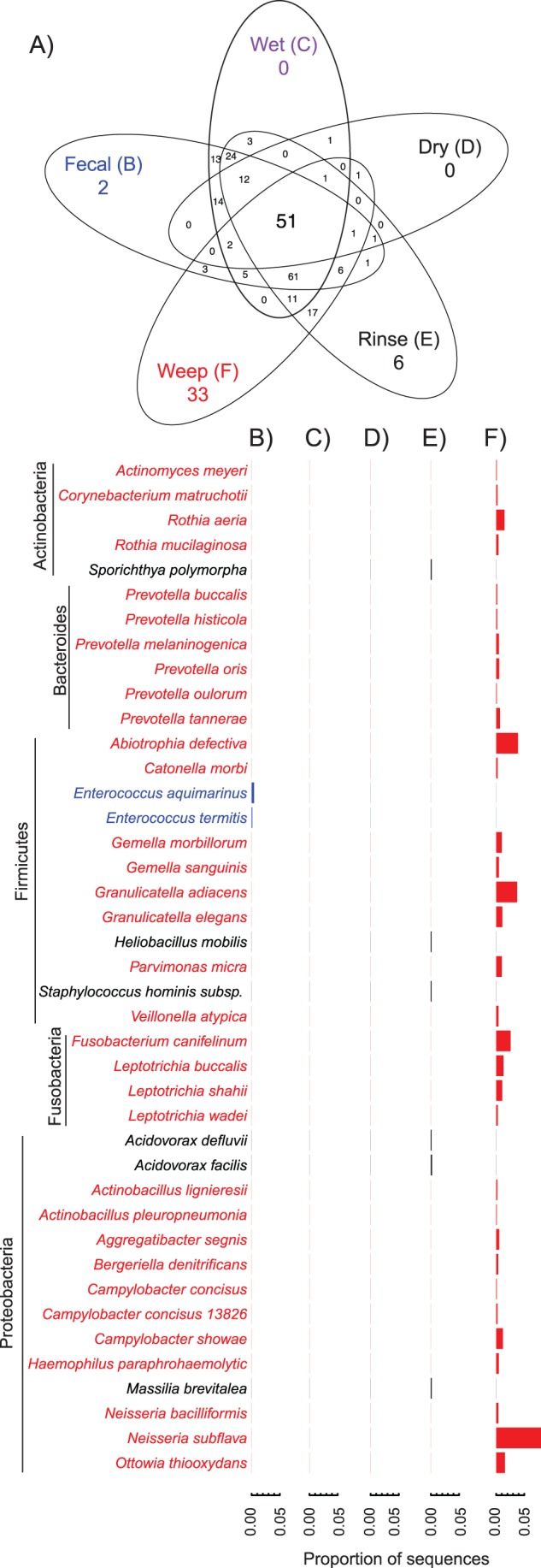
Taxa unique to each sample type as classified to the species level with usearch against the Silva reference database as described in the methods for taxa representing greater than 0.1% of all sequences. A) Number of shared and unique taxa for each of the five sample types shown. The relative abundance of the taxa unique to each sample type is shown for B) Fecal samples (blue), C) Wet litter (none), D) Dry litter (none), E) Carcass Rinses (black), and F) Carcass Weeps (red).

Comparisons of the satellite microbiome – defined as taxa found only in a single sample type – revealed that the most dramatic difference occurred in the carcass weep which had many more unique taxa than expected by chance (p<0.0001; chi-square test). Of 41 taxa accounting for >0.1% of sequences and found only in a single sample type, 33 of these were unique to the carcass weep (Figure 3A–F). Dominant taxa in the weep samples included the anaerobes *Prevotella*, *Veillonella*, and *Leptotrichia* commonly present in the oral microbiome of humans [64], [65] as well as cats and dogs [66], and the aerobe *Neisseria subflava*, shown to dominate experimental biofilms [67]. Of particular relevance to food-safety concerns among taxa uniquely present in the weep were two species of *Campylobacter – C. consisus*, and *C. showae* (Figure 3F). Current dogma generally holds that poultry-associated Campylobacters are *C. jejuni* or *C. coli*, but our results show multiple sequence types present at low relative abundance more closely-related to *C. consisus* and *C. showae* than any known *C. jejuni* sequence. Deep sequencing of the poultry microbiome may challenge existing dogma which is largely based on cultivation with media selective for *C. jejuni* or *C. coli* [8].

The discovery of taxa unique to the carcass weep samples is particularly interesting as, barring contamination from external sources, taxa present at this stage along the farm-to-fork continuum should logically represent a subset of those encountered in other sample types, particularly the carcass rinse samples. Although we can infer from richness estimates that some low-abundance OTUs have not been recovered (Table S1), incomplete sampling does not adequately explain the differences observed for the carcass weep (Figure 3F), as the taxa uniquely present are relatively abundant sequence types (0.1–8% of the total). Given roughly equivalent sampling effort, these taxa should have been encountered in the other sample types had they been present at similar population sizes. Although we did not sample all components of the poultry microbiome (e.g. skin, feathers, etc.), the carcass rinse and weep samples do represent directly paired samples taken from the same 25 carcasses. The relative abundance of taxa in the core microbiome was similar for the rinse and weep samples (Figure 2E–F), but nearly 2/3 of the taxa in the weep samples were not found in the rinse samples collected 48 hr earlier. These sequence types most likely represent taxa which persisted at low abundance through commercial anti-microbial treatment and were able to subsequently multiply during the 48 hr between the two sample collections.

Although it is common for commercial poultry processors in the U.S. to apply antimicrobial chemicals to carcasses during processing (for example, up to 50 ppm sodium hypochlorite in immersion chill tanks; www.fsis.usda.gov/OPPDE/rdad/FSISDirectives/7120.1.pdf), complete sterilization is not the goal and persistence of viable bacterial cells on carcasses following processing has been well documented. For example, cultivable *Salmonella* attached to chicken skin can be recovered after commercial processing [68], and incubations of whole carcasses in enrichment broth can recover bacteria not found by vigorous rinsing of the same carcasses [69]. Sequential carcass rinses have shown that viable bacteria can be continually recovered even after 40 rinses of the same carcass [70], and processed carcasses have been shown to carry the same subtypes of cultivable *C. jejuni* as present in a flock [71]. Our data are consistent with these observations and support a model in which a complex community of viable bacterial cells in and on a carcass can be transferred from the farm into the retail food chain despite current anti-microbial interventions utilized in poultry processing.

#### Detection and quantification of *Clostridium* and *Campylobacter*

To follow specific foodborne pathogens along the food supply chain, we used qPCR to quantify the abundance of two of the most important pathogenic genera, *Campylobacter* and *Clostridium*, across our longitudinal samples using previously validated primer sets [7], [53]. Because of the importance of *C. jejuni* as a human pathogen, we also designed a novel TaqMan qPCR assay specific and sensitive for *C. jejuni* targeting the beta-subunit of the cytolethal distending toxin gene (*cdtB*). CDT is associated with *C. jejuni* pathogenesis, although knowledge of its exact role remains incomplete [72]; here we use it simply as a marker for cdt-positive *C. jejuni*. Of 18 *C. jejuni* genomes in the current version of the IMG database [73], 17 contain at least one cdt gene copy, and phylogenetic analysis of the assay designed here showed it to be highly specific and sensitive (Figure S2).

**Figure 4:**
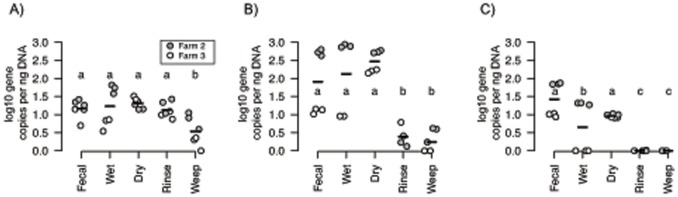
*Clostridium* and *Campylobacter* abundance across sample types as determined by quantitative-PCR for Farm 2 (grey symbols) and Farm 3 (white symbols). Number of 16S rRNA gene copies by sample type and farm for A) *Clostridium* group I assay, and B) *Campylobacter* genus-level-assay, and C) *C. jejuni*-specific cdtB assay. Horizontal bars represent means for sample types for all points within range. Letters denote statistical significance (p<0.05) of pairwise t tests performed in R with the Holm correction for multiple comparisons.

*Clostridium* was detected in all samples, but was significantly reduced (p<0.01, one-sided pairwise t test) in the weep sample relative to the other sample types (Figure 4A). For the genus-level *Campylobacter* assay, inter-farm variability was particularly high for the fecal and wet litter samples with 1.5–2 log differences between the two farms for these sample types. Importantly, significant reductions (p<0.004) were observed for the carcass rinse and carcass weep relative to the on-farm samples for both farms (Figure 4B). For the *C. jejuni*-specific *cdtB* assay, the results were quite similar, with absolute numbers reduced significantly (p<0.05) for the carcass rinse and weep samples compared to the on-farm sample types (Figure 4C). The qPCR results were consistent with the pyrosequencing data in which *Campylobacter* sequence types were only present at very low relative abundance in the rinse and weep samples, and with previous demonstrations of reductions in *C. jejuni* abundance and detection frequency after commercial anti-microbial treatments [74], [75].

### Bacterial Community Associated with Retail Products

**Figure 5:**
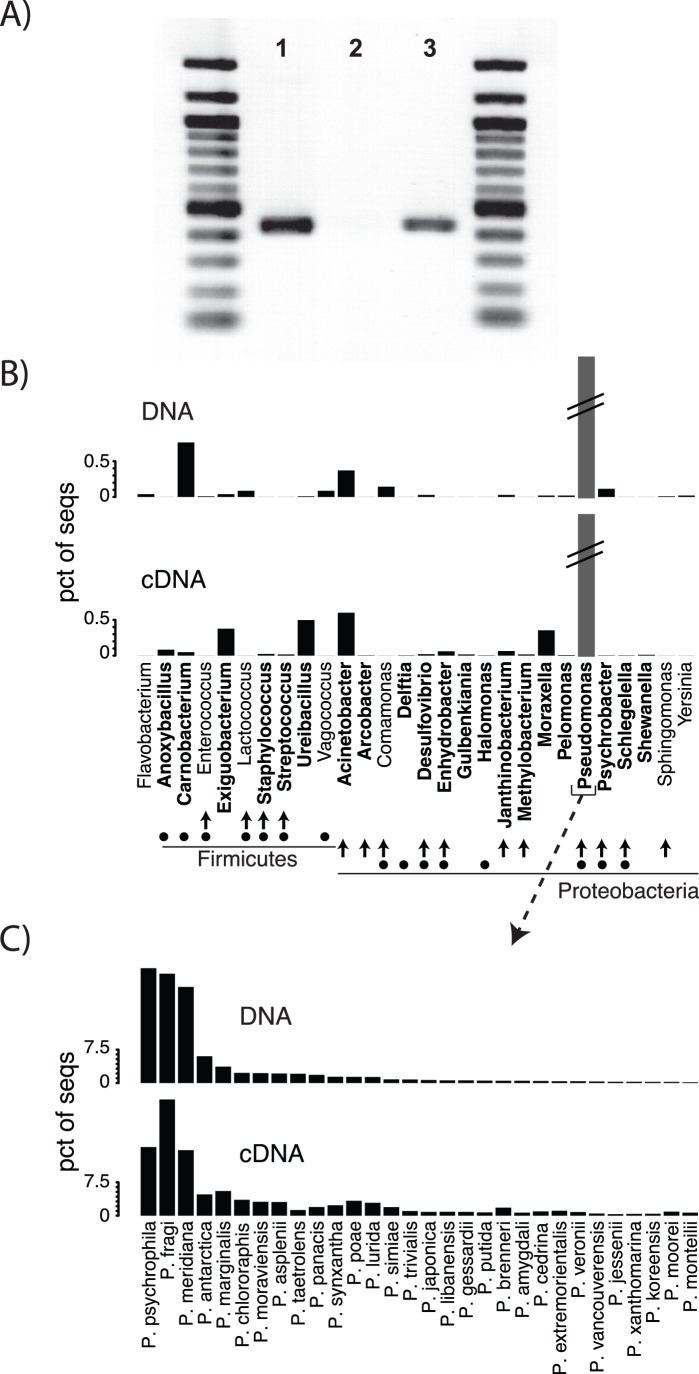
Characterization of bacterial community present in retail poultry products. A) PCR results for amplification from total nucleic acids (lane 1), DNase treated reverse-transcriptase negative control (lane 2), and reverse-transcribed cDNA (lane 3). PCR reactions were performed with the broad-range primers 104F/530R as described in the text. Figure shows pooled products obtained from six retail packages of uncooked chicken parts; MW marker is NEB 100 bp ladder. B) Relative abundance of sequence types recovered from total nucleic acids (upper graph) as compared to sequence types present in the presumed potentially metabolically active fraction of the community (lower graph) classified to the genus level. Genera marked in bold (21 of 28) were designated potentially metabolically active as determined by sequence detection in the cDNA fraction. Genera marked with vertical arrows were also present in the carcass rinse or weep samples of the longitudinal data set. Genera marked with black circles were present in the fecal or litter samples of the longitudinal data set. Note break in Y axis for *Pseudomonas* which comprised 98% of both fractions. C) Comparison of species-level classification of sequences for *Pseudomonas* in DNA (upper graph) and cDNA (lower graph) fractions.

In addition to reductions of *Campylobacter*, decreases in taxonomic richness in the rinse and weep samples demonstrated a community-level effect of residual anti-microbial treatments as applied in the chill tank. However, the uniqueness of the community in the retail weep samples as shown above (Figure 3), strongly suggested that a metabolically active community associated with poultry carcasses can persist through processing and pass into the retail food chain. If this is true, one would expect a complex community of metabolically active bacteria to be associated with poultry products packaged for retail sale. We explicitly tested this hypothesis by purchasing a variety of retail poultry products, extracting RNA from these samples, and performing PCR and 454 sequencing of amplicons generated from the resulting cDNA. Positive amplification from cDNA reverse-transcribed from rRNA with corresponding negative results for controls without reverse-transcriptase confirmed the presence of intact rRNA molecules, presumed to represent potentially metabolically active bacteria (Figure 5A). Following the same data processing steps described above, 10512 sequences were recovered from the DNA fraction, and 25165 sequences were obtained from the cDNA fraction. Taxonomic classification of these sequences showed the presence of 28 different genera in the retail weep fluid, of which 21 were judged potentially metabolically active as determined by sequence recovery from the cDNA fraction (Figure 5B). Interestingly, *Campylobacter* was not detected but the closely-related genus *Arcobacter* was present at low relative abundance in both fractions (Figure 5B). *Campylobacter* has been detected in surveys of retail poultry products at a prevalence of 20–100% of samples, depending on the method of recovery [76]. Of the 28 genera present in the retail weep samples, 20 were also present in the longitudinal data set (Figure 5B). Of the sequences in the retail weep, *Pseudomonas* was by far the most dominant genus, representing 98% of sequences from both the DNA and cDNA fractions (Figure 5B), with a high proportion of sequences most closely related to psychrophilic species such as *P. psychrophila* and *P. antarctica* (Figure 5C). These results are consistent with classification of *Pseudomonas* as a dominant poultry spoilage organism [77], and to our knowledge, represent the first HTS-based characterization of the microbiome associated with retail poultry products.

### Network Analysis

We focused our network analysis on the relationship of *Campylobacter* to co-occurring taxa with several interesting results. First, when classified to the species level by comparison to the Silva reference database, the only significant linkage for any *Campylobacter* taxon was between *C. jejuni* and *C. coli*, neither of which had any significant associations with other taxa (Figure S3). This result was consistent even for relaxed cutoffs for statistical significance and Pearson correlation coefficients. This result may have important implications for *Campylobacter* competitive exclusion (CE) strategies which were first proposed in 1982 [78] but have had only limited success in reducing *Campylobacter* colonization in poultry [79]. If confirmed by additional sequencing of *Campylobacter*-positive samples, the limited efficacy of CE for *Campylobacter* may be explained by a lack of co-occurring taxa, which are presumed to occupy an ecological niche space overlapping with that of *Campylobacter*. To confirm that the inclusion of samples sequenced with the Illumina platform did not influence these results, these samples were excluded from the analysis, with no change in results.

**Figure 6:**
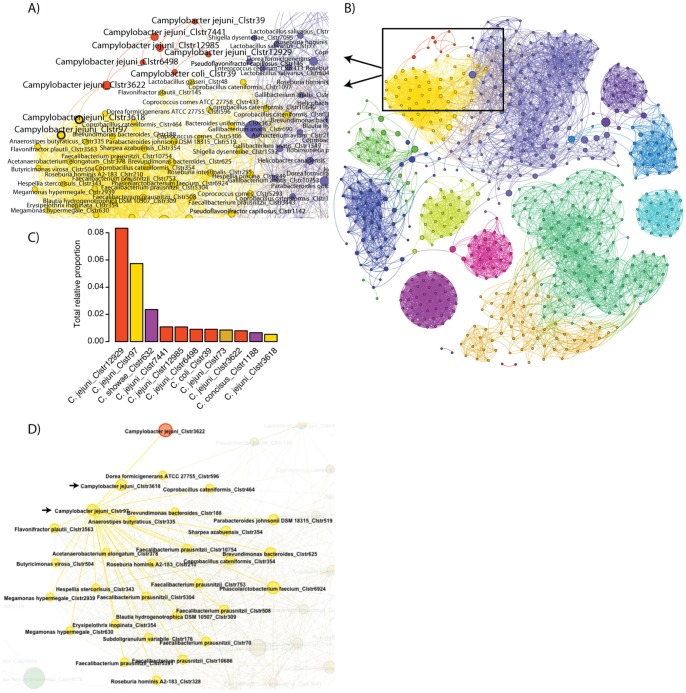
Network analysis of *Campylobacter* OTUs within poultry-associated microbiomes. A) Significant network associations for OTUs classified as *C. jejuni* and *C. coli* shown in detail, and B) in context of entire network. C) *C. jejuni* OTUs with connections only to other *Campylobacter* as shown in orange accounted for a majority of *Campylobacter* sequences. D) Two *Campylobacter* OTUs, Clstr3618 and Clstr97, shown in yellow and denoted by arrows, were connected to a much larger suite of taxa including *Megamonas hypermegale* and *Faecalibacterium prausnitzii* as discussed in the text.

To investigate this question with more discriminatory power, we next performed the same analysis using OTUs mapped to taxonomic classifications. Consistent with the previous analysis, most of the OTUs classified as *Campylobacter* had no significant associations with any other taxonomic group (Figure 6, orange clusters). Interestingly, however, two OTUs, including the 2^nd^ most abundant *Campylobacter* OTU (Clstr97) were significantly co-associated with a suite of other taxa (Figure 6, yellow clusters). Included among these were OTUs most closely related to taxa such as the anaerobe *Faecalibacterium prausnitzii*, known to be abundant in the chicken cecum [59] and the human colon where it produces the short chain fatty acid butyrate and has been shown to employ a flavin-dependent electron transfer scheme to exploit oxic-anoxic boundaries [80]. Another sequence type significantly co-associated with these *Campylobacter* OTUs was *Megamonas hypermegale* which has been previously shown to be associated with *Campylobacter* exclusion in poultry using an elegant antibiotic selection scheme [81]. Interestingly, in this work, Scupham et al. [81] classified *M. hypermegale* sequences as either type I or II and found a significant correlation with *C. jejuni* suppression only from type I sequences. The two *Megamonas* OTUs (Clstr630 and Clstr2939; Figure 6) we found with significant association to *Campylobacter* were clearly more closely related to type II sequences from Scupham et al. (Figure S4). If *Megamonas* type I sequences actually do have a suppressive effect on *C. jejuni*, the predominance of type II sequences associated with *C. jejuni* sequence types in these samples may not be surprising.

Our results are consistent with previous demonstrations of intra-specific diversity of sequence types most closely related to *Campylobacter* [8] and provide further evidence that these sequence types have significantly different ecological niches. Taken together, our network analyses suggest that the limited efficacy historically demonstrated for CE of *Campylobacter* may be due to the variety of ecological niches inhabited by *Campylobacter*. These *Campylobacter* sequence types may represent novel diversity currently undescribed by cultivation; their associations to human illness and food safety remain an interesting and important topic for future research.

### Conclusions

By exploiting the capacities of next-generation sequencing, the approach taken here provides a rapid method to characterize and compare poultry-associated microbial communities independent of biases associated with cultivation. High-throughput sequencing of 16S rRNA gene amplicons has become a common method to investigate microbial communities in complex samples [26], [82], [83], but to our knowledge, this study demonstrates the first use of HTS to characterize the poultry microbiome across a series of farm-to-fork samples.

Analysis of samples along the so-called ‘farm-to-fork’ continuum showed several genera containing recognized pathogens (*Clostridium*, *Campylobacter*, and *Shigella*) belonging to a core microbiome common to all sample types. Quantitative-PCR assays demonstrated that *Clostridium* abundance was significantly reduced in weep samples, while *Campylobacter* generically and *C. jejuni* specifically were significantly reduced in carcass rinse and weep samples relative to fecal and litter samples. The weep samples were the most unique, largely due to the presence of anaerobes such as *Prevotella*, *Veillonella*, *Leptotrichia, C. concisus,* and *C. showae*, suggesting that taxa present at low absolute and relative abundance are able to persist through poultry processing in a viable state. Network analysis revealed that most *Campylobacter* sequence types do not have significant associations with other taxa which may explain the historically poor efficacy of attempts to competitively exclude *Campylobacter*. Two *C. jejuni* sequence types did have significant linkages to a suite of other taxa, including *Megamonas hypermagale*, consistent with previous observations [81]. High-throughput sequencing provides a powerful tool to identify potential reservoirs of foodborne pathogens and analyse interactions within complex microbial communities.

## Supporting Information

Figure S1 **Rarefaction curves by sample for OTU designations at 97%, 95%, and 90% similarity cutoffs defined with uclust as described in the text.**
(EPS)

Figure S2 **Taxonomic specificity and sensitivity of cdtB TaqMan qPCR assay.** Maximum-likelihood tree contains all non-redundant publicly available sequences from the IMG database retrieved by annotations of ‘cdt’, ‘cytolethal distending toxin’, and IMG genome blast with default parameters. Sequences with perfect matches to the newly-designed assay are shown in red; all other sequences have >5 mismatches to each primer and probe.
(EPS)

Figure S3 **Species level network analysis showing significant associations for Campylobacter only between C. coli and C. jejuni as shown in boxed area.**
(EPS)

Figure S4 **Representative sequences classified as** ***Megamonas*** **recovered by our sequencing compared to** ***Megamonas*** **sequences in Scupham et al. (2010) shown in bold.** All sequences found here were classified as cluster II, not cluster I which were found to be significantly associated with *C. jejuni* suppression by Scupham et al. (2010).
(EPS)

Table S1 **Sample sources, and taxonomic richness and diversity observed at a 95% clustering level for samples sequenced in this study.**
(DOCX)
