# Supplementary material for: The Poultry-Associated Microbiome: Network Analysis and Farm-to-Fork Characterizations
Source: PLoS One. 2013 Feb 27;8(2):e57190. doi: 10.1371/journal.pone.0057190 (PMC3584146; doi:10.1371/journal.pone.0057190)
Supplement: Table S1 — Sample sources, and taxonomic richness and diversity observed at a 95% clustering level for samples sequenced in this study. (DOCX) [file pone.0057190.s005.docx]

Supplementary Table S1. Sample sources, and taxonomic richness and diversity observed at a 95% clustering level for samples sequenced in this study. Each flock represented ca. 16,000 birds.

| **Sample** | **Source** | **Level of sampling** | **Richness** | **Shannon** | **Chao1** | **se. Chao1** | **Original description** |
| --- | --- | --- | --- | --- | --- | --- | --- |
| F2-Dry | Litter | Flock | 36 | 0.637 | 66 | 44 | this study |
| F2-Fecal | Feces | Flock | 727 | 4.045 | 1422 | 104 | this study |
| F2-Rinse | Rinse | 25 birds | 446 | 4.792 | 626 | 37 | this study |
| F2-Weep | Weep | 25 birds | 365 | 4.118 | 506 | 33 | this study |
| F2-Wet | Litter | Flock | 907 | 5.008 | 1488 | 78 | this study |
| F3-Dry | Litter | Flock | 443 | 4.055 | 1020 | 107 | this study |
| F3-Fecal | Feces | Flock | 2104 | 5.003 | 4979 | 246 | this study |
| F3-Rinse | Rinse | 25 birds | 715 | 5.104 | 1550 | 125 | this study |
| F3-Weep | Weep | 25 birds | 487 | 4.575 | 1253 | 148 | this study |
| F3-Wet | Litter | Flock | 895 | 5.196 | 2716 | 239 | this study |
| Retail Weep (DNA) | Weep | Multiple flocks | 49 | 0.77 | 61 | 4 | this study |
| Retail Weep (cDNA) | Weep | Multiple flocks | 44 | 0.77 | 50 | 3 | this study |
| G3B | Cecum | 1 bird | 1524 | 3.672 | 4738 | 321 | [[1](#_ENREF_1)] |
| G6B | Cecum | 1 bird | 1647 | 3.746 | 5046 | 324 | [[1](#_ENREF_1)] |
| PED12 | Rinse | Flock | 869 | 5.519 | 1317 | 66 | [[2](#_ENREF_2)] |
| PED34 | Rinse | Flock | 1157 | 5.875 | 1661 | 65 | [[2](#_ENREF_2)] |
| R10 | Rinse | 1 bird | 890 | 5.741 | 1371 | 66 | [[2](#_ENREF_2)] |
| R2 | Rinse | 1 bird | 1134 | 6.085 | 1541 | 55 | [[2](#_ENREF_2)] |
| R6 | Rinse | 1 bird | 910 | 5.807 | 1472 | 76 | [[2](#_ENREF_2)] |
| WK1-DF1-5 | Cecum | 5 birds | 2194 | 4.749 | 7169 | 417 | [[3](#_ENREF_3)] |
| WK1C-6-10 | Cecum | 5 birds | 2325 | 4.646 | 6966 | 379 | [[3](#_ENREF_3)] |
| WK2-DF1-5 | Cecum | 5 birds | 1887 | 4.607 | 5760 | 348 | [[3](#_ENREF_3)] |
| WK2-DF6-10 | Cecum | 5 birds | 2397 | 5.213 | 7333 | 389 | [[3](#_ENREF_3)] |
| WK2C-6-10 | Cecum | 5 birds | 1974 | 4.844 | 6053 | 365 | [[3](#_ENREF_3)] |
| WK3C-6-10 | Cecum | 5 birds | 2116 | 5.056 | 6294 | 352 | [[3](#_ENREF_3)] |
| Wk3DF1-5C | Cecum | 5 birds | 644 | 5.481 | 1132 | 74 | [[3](#_ENREF_3)] |

1. Tillman GE, Haas GJ, Wise MG, Oakley B, Smith MA, et al. (2011) Chicken intestine microbiota following the administration of lupulone, a hop-based antimicrobial. FEMS Microbiol Ecol.

2. Line JE, Oakley BB, Stern NJ (In Press) Comparison of cumulative drip sampling to whole carcass rinses for estimation of *Campylobacter* spp. and quality indicator organisms associated with processed broiler chickens. Poultry Science.

3. Wise MG, Siragusa GR (2007) Quantitative analysis of the intestinal bacterial community in one- to three-week-old commercially reared broiler chickens fed conventional or antibiotic-free vegetable-based diets. J Appl Microbiol 102: 1138-1149.
